# Kidney-Replenishing Herb Induces SOCS-3 Expression via ERK/MAPK Pathway and Improves Growth of the First-Trimester Human Trophoblast Cells

**DOI:** 10.1155/2017/2473431

**Published:** 2017-09-28

**Authors:** Chang-ying Xing, De-xiang Zhang, Sui-qi Gui, Min-fang Tao

**Affiliations:** ^1^Shanghai Jiao Tong University Affiliated Sixth People's Hospital, Shanghai 200233, China; ^2^Affiliated Hospital of Nantong University, Nantong 226001, China; ^3^The Hospital of Obstetrics & Gynecology, Shanghai 200011, China

## Abstract

Kidney-replenishing herb is a traditional medicine formula in China which has been widely used for clinical treatment of recurrent miscarriage. Our previous study showed that Kidney-replenishing herb could promote proliferation and inhibit apoptosis of the human first-trimester trophoblasts. In the present study, we further explored the potential mechanism and signal pathway of Kidney-replenishing herb on human trophoblast cells. Our research showed that Kidney-replenishing herb stimulated proliferation and reduced apoptosis of human trophoblast cells in vitro, and this appeared to be positive correlation with SOCS-3 transcription, suggesting that Kidney-replenishing herb regulated biological functions of human trophoblast cells by inducing SCOS-3 expression. Furthermore, the Kidney-replenishing herb treatment stimulated the phosphorylation of ERK1/2, and blocking the signaling pathway by mitogen-activated protein MAPK (MEK) inhibitor, U0126, inhibited Kidney-replenishing herb-induced SOCS-3 transcription, depressed proliferation, and promoted apoptosis of human trophoblasts. Kidney-replenishing herbs still induced ERK1/2 phosphorylation after SOCS-3 siRNA silence. Overexpression of SOCS-3 stimulated the proliferation of trophoblast. These findings suggest that SOCS-3 expression is induced by Kidney-replenishing herbs via activation of MAPK pathways, and this may possibly be involved in promoting human trophoblast cells growth which is contributed to embryo development.

## 1. Introduction

A successful pregnancy requires perfect placenta conditions. The early placental development is defined by an intricate balance between cellular proliferation and apoptosis of trophoblast cells [1, 2]. Proliferation markers are strongly expressed in cytotrophoblast in early stages of gestation [3]. At the same time, apoptosis is present in trophoblast cells throughout gestation and is believed to be physiologically important for normal placental development and fetal growth [4]. It is well accepted that the insufficient trophoblast growth or excessive apoptosis occurs in the placenta of human first-trimester spontaneous pregnancy loss and fetal growth restriction. Our previous studies demonstrated the positive influence of Kidney-replenishing herb on the proliferation and inhibition of IFN-*γ* induced apoptosis of human first-trimester trophoblasts [5]. However, the roles of Kidney-replenishing herb in regulation of trophoblast cells function and relative molecular mechanisms remain unclear.

The extracellular signal-regulated kinase 1/2 (ERK1/2)/mitogen-activated protein kinase (MAPK) pathway is one of the important signaling cascades which is involved in the cell proliferation and embryo development; furthermore ERK1/2 MAPK signal transduction also provides protection against apoptosis in several cell types when they are challenged by cellular stresses or chemotherapeutic drugs [6]. ERK1/2 is widely expressed and markedly activated in the villous cytotrophoblasts throughout early embryonic development [7]. Mice embryos which lacked ERK2 died early during embryogenesis because of the defection in trophoblast development. Mutant embryos fail to form the ectoplacental cone and extraembryonic ectoderm, which derives from the polar trophectoderm [8]. These indicate that ERK2 is required for the proliferation of trophoblast stem cells in the polar trophectoderm, and ERK1/2/MAPK pathway has an important function in the regulation of trophoblasts growth.

Suppressors of cytokine signaling (SOCS) protein are a family of intracellular proteins that control cytokine signaling [9]. The SOCS family consists of at least eight members, including cytokine induced SH2 protein (CIS) and SOCS-1–7. SOCS proteins contain a central Src homology 2 domain protein (SH2) domain, a conserved C-terminus referred to as the SOCS box, and unique N-terminal region [10]. SOCS family proteins can be induced by cytokines, growth factors, and several immunomodulators. Roberts and colleagues showed that mice with a deletion of SOCS-3 gene died at midgestation because of the placental defects. They observed that SOCS-3(−/−) embryos were slightly smaller than the wild type but appeared otherwise normal. However, the placental spongiotrophoblast layer was significantly reduced and accompanied by increased numbers of giant trophoblast cells. The network of embryonic vessels and maternal sinuses was inadequately developed, and yolk sac erythropoiesis was normal. They concluded that the embryonic lethality is not caused by anatomical defects of the embryo, but rather poor placental development that results in the embryo developmental arrest and death [11]. Therefore, it is believed that SOCS-3 is critical for a successful pregnancy outcome by regulating trophoblast function during the placental development. An interesting study reported by Isobe and colleagues showed that MEK antagonist could inhibit SOCS-3 expression of the undifferentiated rat trophoblast-like cell line [12]. Another study has demonstrated that SOCS-3 binds and inactivates RasGAP, a negative regulator of Ras signaling, leading to increased Ras/MAPK pathway activity in human JEG-3 trophoblastic cells [13]. These data suggest that the activation of ERK1/2/MAPK pathway signal pathway may be related to SOCS-3 expression. However, the roles of SOCS-3 in the induction of proliferation processes and relative molecular mechanisms in primary first-trimester human trophoblasts are still unclear.

In this study, we observed the effects of Kidney-replenishing herbs on the proliferation and apoptosis of human first-trimester trophoblasts; we therefore investigated the effects of the herbs on the expression of SOCS-3 in human trophoblasts by reverse transcription-polymerase chain reaction (RT-PCR) and Western blot. In order to elucidate the intracellular signal pathway mediating the upregulation in SOCS-3 expression by Kidney-replenishing herb, we investigated the phosphorylation of ERK1/2 signal pathway involved in the proliferative and apoptosis of human trophoblasts by Kidney-replenishing herb.

## 2. Materials and Methods

### 2.1. Isolation and Cultivation of Human First-Trimester Cytotrophoblast

First-trimester human villous tissues (5–10 weeks of gestation) were collected from clinically normal pregnancies which were terminated for nonmedical reasons, at the Hospital of Obstetrics and Gynecology, Fudan University Shanghai Medical College. The study was approved by the Fudan University Ethical Review Board, and each patient signed the consent form. Villous tissues were immediately suspended in ice-cold DMEM and transmitted to the laboratory, where they were washed 2-3 times in sterile phosphate-buffered saline (PBS) to remove the excess blood. Primary trophoblast cells were isolated by trypsin-DNase type I digestion and layered over a discontinuous Percoll gradient, as described by our previous study [14]. This assay supplies a 95% purity of trophoblast cells. These isolated human trophoblast cells and the choriocarcinoma JAR cell line were cultured in DMEM supplemented with 2 mM glutamine, 10% FCS at 37°C in 5% CO_2_.

### 2.2. Preparation of Serum

Kidney-replenishing herbs are traditional medicines which have satisfactory effects on threatened miscarriages, including* Dangshen* 12 g,* Tusizi* 15 g,* Baishu* 6 g,* Baishao* 9 g,* Duzhong* 12 g,* Sangjisheng* 12 g,* Sugeng* 6 g, and* Tiaohuangqin* 15 g. Herb serum was made according to our previous study [14]. All of the herbs were collected and made by the scholars of Shanghai Graduate School of Medication (Chinese Academy of Science).

### 2.3. Materials

Histochemical ABC kit was purchased from Sino-American Bio-Technology (Luoyang China). RevertAid™ First Strand cDNA Synthesis Kit and* Taq* DNA polymerase were purchased from Fermentas (Thermo Fisher Scientific, MA, USA). BCA-100 Protein Quantitative Analysis Kit was purchased from Sangon Biotechnology (Shanghai, China). Mouse anti-GAPDH monoclonal antibodies and plasmid extraction kit were purchased from Kangcheng Biotechnology (Shanghai, China). Rabbit anti-human SOCS-3 antibodies, mouse anti-phosphoERK monoclonal antibodies, rabbit anti-ERK monoclonal antibodies, and mouse anti-GAPDH were purchased from Santa Cruz Biotechnology (Santa Cruz, CA, USA). Annexin V-FITC apoptosis kit, PRK5 plasmid, and pRNAT-U6.1 plasmid were purchased from R&D Systems (Minneapolis, MN, USA). HRP-goat anti-rabbit IgG was purchased from Dingguo Biotechnology (Shanghai, China). TRIzol reagent, BLOCK-iT™ oligo [13750062], and Lipofectamine™ 2000 Kit were purchased from Invitrogen (Carlsbad, CA, USA). SOCS-3 forward primer (5′-CGCCTCAAGACCTTCAGCTC-3′) and reverse primer (5′-ATCCAGGAACTCCCGAA-3′), product 650 bp, GAPDH forward primer (5′-GAAGGTGAAGGTCGGAGTC-3′) and reverse primer (5′-GATGGTGATGGGATTTC-3′), and product 220 bp were designed and integrated by Kangcheng Biotechnology (Shanghai, China).

### 2.4. RT-PCR Analysis of SOCS-3 Expression in Human First-Trimester Trophoblast

Total cellular RNA ofhuman first-trimester trophoblast was extracted using TRIzol reagent and cDNA Synthesis Kit according to the manufacturer's protocol. The total RNA of 3 *μ*g was denatured, and RT was performed for 1 h at 42°C with 0.5 mg Oligo dT 0.5 *μ*g, 10 mM dNTP 2 *μ*l, RNase inhibitor 1 *μ*l, 200 U Moloney virus-reverse transcriptase, and 10 reaction buffer in a total volume of 20 ml. After 5 min precycle at 95°C, 30 cycles of denaturation at 94°C (50 s), the reaction was followed by 30 cycles of 50 s at 55°C and 50 s at 72°C. When the final cycle was over, samples were kept at 72°C for 15 min for the further extension. The PCR products were separated using a 2% agarose gel and ethidium bromide-stained bands were photographed. The relative intensity of SOCS-3 is the ratio of the absorbance value of SOCS-3 to that of GAPDH.

### 2.5. Western Blot

Cells were harvested and lysed for 30 min in 1 ml of RIPA buffer (50 mM Tris–HCl, pH 7.4, 150 mM NaCl, 1% NP-40, 10 mM NaF, 0.25% sodium deoxycholate, 1 mM EDTA, 1 mM PMSF, and phosphatase inhibitors) and 10 *μ*l cocktail; the extracts were incubated for 20 min on ice and cleared by centrifugation. Samples were incubated in SDS-PAGE (SDS-polyacrylamide gel electrophoresis) sample buffer at 95°C for 10 min. The proteins were separated in a gel containing 10% acrylamide. After that, the separated proteins were transferred onto PVDF membrane. The membranes were incubated with 5% nonfat milk powder in Tris-buffered saline (0.5 M Tris, 1.5 M NaCl) supplemented with 0.1% Tween (TBST) for 1 h. Incubation overnight at 4°C with the primary antibody SOCS-3 diluted 1 : 500 for detection of SOCS-3, anti-GAPDH diluted 1 : 5000 for detection of GAPDH, anti-ERK1/2 diluted 1 : 1500 for detection of ERK1/2, and antiphosphorylated ERK1/2 diluted 1 : 1000 for detection of phosphorylated ERK1/2. After washing with TBST three times, the membrane was incubated with horseradish peroxidase secondary antibodies at a dilution of 1 : 2000 in 1% BSA-TBST at 37°C for 1 h. The proteins were detected using ECL Western blotting detection reagents.

### 2.6. Immunohistochemistry for SOCS-3 in Villous Tissue

Paraffin-embedded sections of human first-trimester placental bed samples were deparaffinized, rehydrated, and antigen retrieved. Endogenous peroxides activity was quenched with 3% H_2_O_2_. Samples were covered with normal blocker serum and incubated with rabbit anti-human SOCS-3 antibody at 1 : 100 dilution. The sections were then treated with appropriate avidin-biotin histostain kit according to the manufacturer's instructions. Slides were stained with DAB and counterstained with haematoxylin. The isotype-matched control antibodies were used to exclude nonspecific staining.

### 2.7. Flow Cytometry (FCM)

FCM was applied to measured cells proliferation. Human trophoblasts were harvested and fixed 30 min with 70% ethanol at 4°C and then resuspended in 0.5 ml of PBS containing PE-PCNA mouse anti-human antibody (20 *μ*l/10^6^ cells) incubated at 37°C for 30 min. A total of 20,000 cells were routinely acquired in a FACScan flow cytometer (BD Biosciences, CA, USA).

In order to measure cells apoptosis, the trophoblast cells were treated by Kidney-replenishing herb serum and control serum and then washed with PBS in binding buffer at a density of 4 × 10^5^ cells (10 Mm Hepes, pH 7.4, 140 mM NaCl, and 2.5 mM CaCl2). Fluorescein isothiocyanate (FITC)-Annexin V and PI were added to a final concentration of 1 *μ*g/ml. The mixture was incubated for 10 min and then analyzed by FCM as described above.

### 2.8. Detection of Cell Viability

The trophoblast cells were plated in a 96-well plates at a density of 2 × 10^4^ cells per well. After cells were treated with a series of concentrations of Kidney-replenishing herb for 48 h and incubated at 37°C, MTT (5 mg/ml) 20 *μ*l was added to the cells. After 4 h incubation, the absorbance was measured with a wavelength of 450 nm.

### 2.9. SOCS-3 Plasmid Transfection

The SOCS-3-PRK5 plasmid construct was provided by Dr. Miura Osamu, from Tokyo Medical and Dental University. JAR choriocarcinoma cell line was recognized a model to study placental functions. JAR cells were seeded in a six-well culture plate of 2 × 10^4^/well. When cells reached 80% confluent, they were transfected with SOCS-3 expression plasmids using Lipofectamine 2000 plus reagent according to the manufacturer's instructions. PRK5 were used as a control. Successful transfection of SOCS-3 genes into trophoblasts was confirmed by positive green fluorescence reviewed under fluorescent microscope. After 6 h of incubation, cells were incubated with DMEM containing 10% FBS for 48 h, their mRNA expression detected by RT-PCR, and protein expression by SDS-PAGE.

### 2.10. SOCS-3 Stealth™ siRNA Transfection

SOCS-3 Stealth siRNA was procured from Invitrogen. The siRNA sequence targeting SOCS-3 is 5′-AGUAGAUGUAAUAGGCUCUUCUGGG-3′. A random siRNA (5′-CCCAGAAGAGCCUAU-3′) which does not have any target region in human genes served as negative control. JAR cells were seeded in a six-well plate of 2 × 10^4^/well. When cells reached 80% confluent, medium was changed to OPTIMEM. The siRNA oligonucleotides targeting NME1 (Invitrogen, Carlsbad, CA) and Lipofectamine 2000 were mixed in OPTIMEM and then added to the cells at room temperature. Efficiency of silencing of the target gene was assessed by Western blot after transfection 72 h.

### 2.11. Data Analysis

Data were expressed as the mean ± SD. Data were analyzed with application of the two-way ANOVA using the SPSS 13.0 software package; differences were considered as statistically significant at *p* < 0.05.

## 3. Results

### 3.1. Kidney-Replenishing Herb Enhanced Proliferation of Human First-Trimester Trophoblasts and JAR Cell Lines

**Figure 1:**
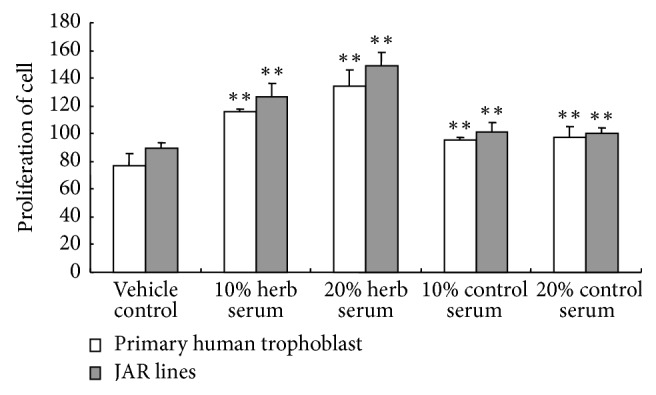
Kidney-replenishing herb promoted human first-trimester trophoblast proliferation activity. We first evaluated the modulation of Kidney-replenishing herb on proliferation activity of human trophoblast by MTT, as shown in this figure; within 10–20% ranges of concentrations, Kidney-replenishing herb stimulated the trophoblast proliferation in a dose-dependent manner compared control. Likewise, Kidney-replenishing herb affected JAR cell proliferation in a similar manner. ^*∗∗*^*p* < 0.01 versus the vehicle control.

To testify the effects of Kidney-replenishing herb on the proliferation of human trophoblast, we first explored its effect on cell viability. Primary human trophoblasts and JAR were cultured in DMEM with 1% FBS for 12 h and then cultured in DMEM with 10% and 20% of Kidney-replenishing herb for 48 h, while cells were simultaneously treated with rat serum (10%, 20%) for a duration of 48 h as a positive control. Analysis of the demographic data revealed that control serum enhanced cell viability compared with vehicle control group (*p* < 0.01) and suggested that serum alone stimulated cell viability, but there is no significant difference in different concentration of control serum. As shown in Figure 1, the proliferation of cells was significantly higher in Kidney-replenishing herb compared with the same concentration control serum (*p* < 0.01). 20% of Kidney-replenishing herb activated cell viability higher than that of 10% Kidney-replenishing herb exposure (*p* < 0.05). Kidney-replenishing herb promoted the trophoblast proliferation in a dose-dependent manner. Likewise, Kidney-replenishing herb stimulated JAR cell proliferation in a similar manner.

### 3.2. Kidney-Replenishing Herb Promoted Growth of Human First-Trimester Trophoblast Cells through MAPK/ERK1/2 Signaling Pathway

MAPK/ERK1/2 signaling pathway is involved in regulation of proliferation and apoptosis of human trophoblasts; we wondered whether Kidney-replenishing herb improved the growth of human first-trimester trophoblasts via MAPK/ERK1/2 signaling pathway or not.

**Figure 2:**
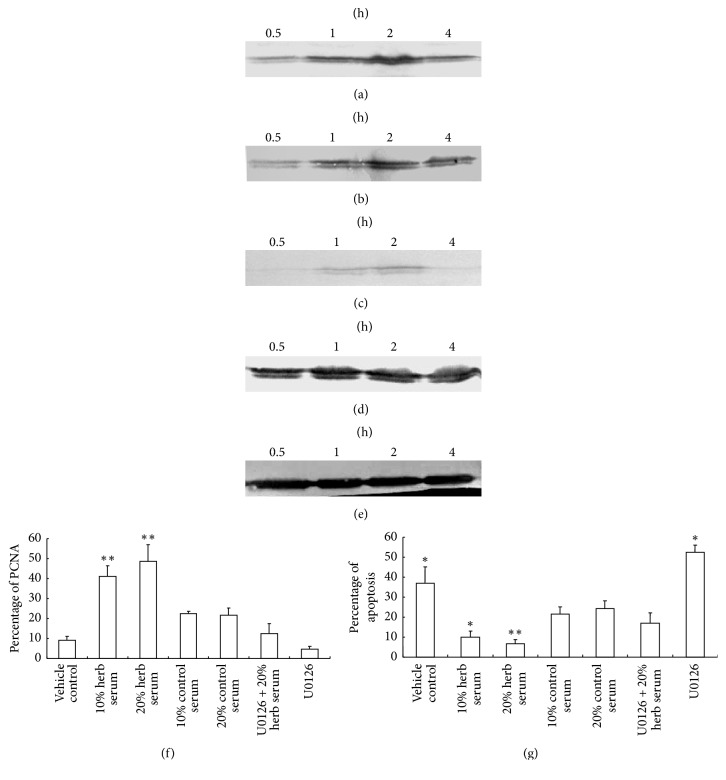
Modulation of Kidney-replenishing herb on growth of the primary cultured human first-trimester trophoblast cells. To verify rapid activation of ERK1/2, cells were starved for 12 h, subjected to 10% or 20% of Kidney-replenishing herb treatments for 4 h. The phosphorylation of ERK1/2 was observed in response to Kidney-replenishing herb treatment ((a), (b)). The phosphorylation of ERK1/2 induced by 20% of control serum was also detected (c). The samples were controlled by immunoblot against ERK (d) and GAPDH (e). The primary cultured trophoblasts were starved with 1% FBS for 12 h and then treated with vehicle, 10% Kidney-replenishing herb, 20% Kidney-replenishing herb, 10% control serum, 20% control serum, and Kidney-replenishing herb combined with U0126 (30 mmol/l) or U0126 (30 mmol/l) alone, respectively, for 48 h. The percent of PCNA expression (f) or Annexin-V-FITC/PI staining (g) was observed by flow cytometry. ^*∗*^*p* < 0.05, ^*∗∗*^*p* < 0.01 when compared with 20% control serum.

After being incubated in DMEM with 1% FBS for 12 h, cells were treated with 10% or 20% of kidney-replenishing herb for 0.5, 1, 2, and 4 h to analyze the phosphorylation of ERK1/2 by Western blot analysis. The results in Figures 2(a) and 2(b) showed that Kidney-replenishing herb stimulation evoked dose-dependent ERK1/2 phosphorylation, and the activation persisted for at least 4 hours, the maximal effect was achieved at 2 h. Otherwise, the slight effect was also observed at 20% of control serum; as shown in Figure 2(c), it suggested that serum could also induce trophoblasts ERK1/2 phosphorylation. U0126, a specific inhibitor of ERK upstream kinase MEK1/2, almost significantly inhibited activation of ERK induced by Kidney-replenishing herb. It could be concluded that Kidney-replenishing herb activated the ERK1/2/MAPK signaling pathway in human trophoblast cells.

To test the effects of Kidney-replenishing herb on human trophoblast cell proliferation, the flow cytometry was carried out to measure PCNA. We cultured cells in the serum of concentrations of Kidney-replenishing herb (0, 10, and 20%) for 48 h, the same concentration of control serum (10, 20%) as a control. At the same time, we treated cells with absence or presence of 20% herb in the presence of 30 *μ*mol/L U0126. As shown in Figure 2(f), Kidney-replenishing herb substantially increased proliferation of human first-trimester trophoblast cells in a dose-responsive manner, the proliferation of human first-trimester trophoblast cells was significantly higher with Kidney-replenishing herb concentrations of 10% and 20% (*p* < 0.01) compared with either concentration control serum, and this proliferation reached a peak in the 20% of Kidney-replenishing herb. Combined with the results of the above MTT and our other study [8], it suggested that Kidney-replenishing herb can stimulate proliferation of the first-trimester trophoblast cells in an appropriate concentration range by inducing alterations in both cell viability and morphology. After treatment with U0126, cell proliferation was inhibited almost 80%, and U0126 repressed the herb effect on the proliferation relative to that of 20% of herb (*p* < 0.01). Our results suggested that ERK1/2 pathway was involved in Kidney-replenishing herb regulation of trophoblast proliferation.

In addition, we measured the early apoptotic events by simultaneous detection of Annexin-V-FITC/PI staining using flow cytometry. As shown in Figure 2(g), control serum group inhibited apoptoticcell to a certain degree compared with vehicle control (*p* < 0.05), but there was no significant difference between different concentration of control serum. Kidney-replenishing herb repressed the apoptotic effect on the U0126 relative to that vehicle control (*p* < 0.01). Furthermore, Kidney-replenishing herb prevented apoptosis of trophoblastic cells compared with the same concentration control serum (*p* < 0.05). It suggested that Kidney-replenishing herb prevents early apoptotic events in trophoblastic cells by activating ERK1/2 pathway.

### 3.3. Human First-Trimester Trophoblasts Expressed SOCS-3 In Vivo

**Figure 3:**
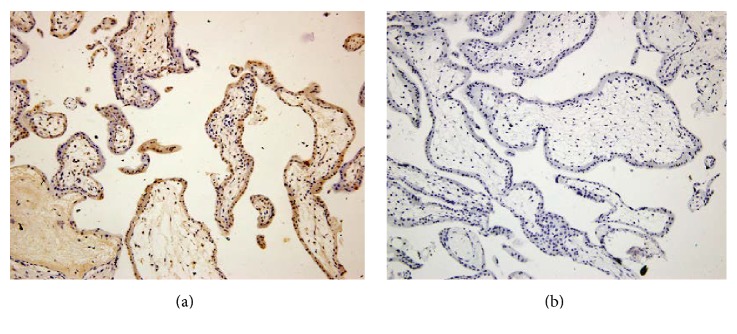
SOCS-3 expression in vivo in human first-trimester placental bed tissue. Specific brown-coloured staining for SOCS-3 was detected in the cytoplasm and nucleus of villous trophoblasts (a). No background staining was observed in the isotype control experiments (b). Magnification ×200.

We detected SOCS-3 protein expression in human first-trimester villus by immunohistochemistry. SOCS-3 was expressed in the nucleus and cytoplasm of human trophoblast cells, as shown in Figure 3. Immunohistochemical procedures were performed two times and similar results were obtained.

**Figure 4:**
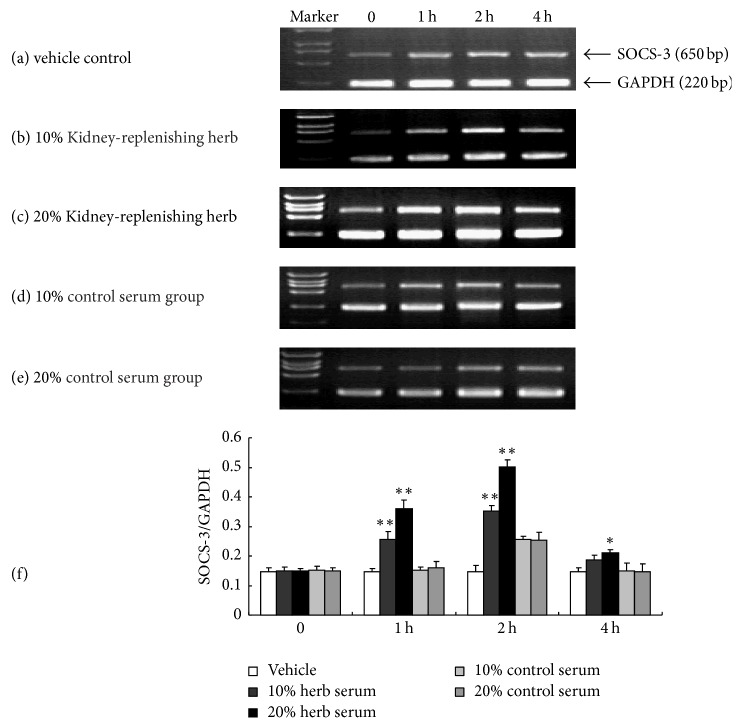
SOCS-3 transcription in the Kidney-replenishing herb-treated human first-trimester trophoblast. As shown in (a), there were weak SOCS-3 transcription in group of vehicle control and no increased expression of SOCS-3 within 4 h. Then we further observed the transcription of SOCS-3 in the primary cultured first-trimester trophoblast cells after treatment with 10% (b) or 20% (c) Kidney-replenishing herb for different times. The slight SOCS-3 mRNA amount was determined in human trophoblasts response to treatment by 10% and 20% group of serum control at 2 h treatment ((d), (e)). We founded that SOCS-3 transcription increased slightly after Kidney-replenishing herb treatment for 1 h and reached its peak at 2 h, and Kidney-replenishing herb increased the transcription of SOCS-3 in trophoblast cells in a dose-dependent manner (f). ^*∗∗*^*p* < 0.01, ^*∗*^*p* < 0.05 versus 20% control serum.

To study the influence of Kidney-replenishing herb on the expression of SOCS-3 in human trophoblasts, the cells were cultured in DMEM with 1% FBS for 12 h and then treated with 0, 10, and 20% Kidney-replenishing herb and 10% and 20% control serum. We detected dim SOCS-3 transcription in vehicle control group (Figure 4(a)), which suggested little endogenous SOCS-3 expression in primary cultured human trophoblasts, 10% or 20% serum induced SOCS-3 expression at 2 hours' stimulation (Figures 4(d) and 4(e)). However, there is no increased expression of SOCS-3 between treatment by 10% and 20% group of control serum (*p* > 0.05), suggesting that SOCS-3 may be transient reduced by serum. Meantime, we found that SOCS-3 was upregulated at significant levels only 1 h after Kidney-replenishing herb administration (Figures 4(b) and 4(c)), then was enhanced rapidly, reached its peak at 2 h, and was slowly declined, but after 4 h of treatment, it was still above the basal level. As shown in Figure 4(f), treatment with various concentrations of Kidney-replenishing herb increased the transcription of SOCS-3 in trophoblast cells in a dose-dependent manner. There is a statistically significant SOCS-3 transcription which increased in various concentrations of Kidney-replenishing herb group compared with the either control serum group or vehicle group (*p* < 0.01).

### 3.4. Kidney-Replenishing Herb Enhanced SOCS-3 Protein Expression In Vitro in Human First-Trimester Trophoblasts

**Figure 5:**
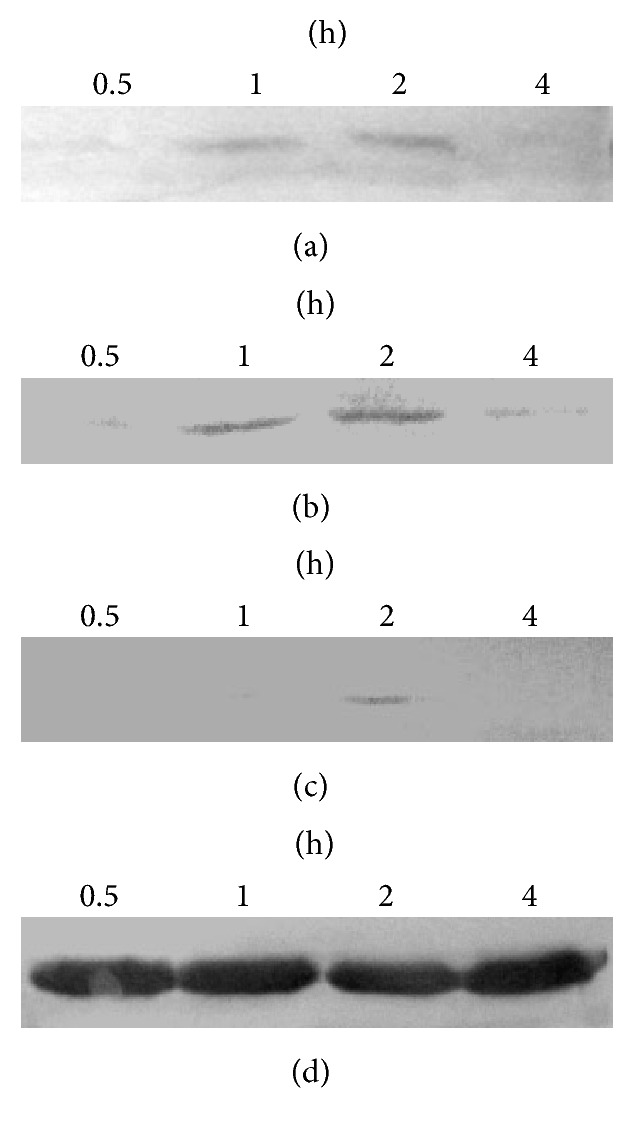
Kidney-replenishing herb promoted SOCS-3 protein expression in human first-trimester trophoblasts. Primary human trophoblasts were incubated for 4 h with 10% to 20% Kidney-replenishing herb. As shown in (a), (b) there is also an increase of SOCS-3 protein expression (0.5, 1, 2, and 4 h). Control cells were treated with serum in the same way without herb and detected a trace SOCS-3 expression at 2 h (c). The levels of GAPDH were examined as an internal control (d). The experiments were performed at least three times and representative result was presented.

After observing increased SOCS-3 transcription in the first-trimester human trophoblast cells treated with Kidney-replenishing herb, we analyzed SOCS-3 protein expression in trophoblast cells cultured for 4 h by Western blot. As shown in Figure 5(c), there appeared SCOS3 band in the serum treated primary human trophoblasts, and the protein levels of SOCS-3 was significantly increased after treatment with Kidney-replenishing herb (Figures 5(a) and 5(b)), suggesting that SOCS-3 was successfully induced by Kidney-replenishing herb in human trophoblast cells.

### 3.5. U0126 Influence on SOCS-3 Expression in the Kidney-Replenishing Herb-Treated Human First-Trimester Trophoblast

**Figure 6:**
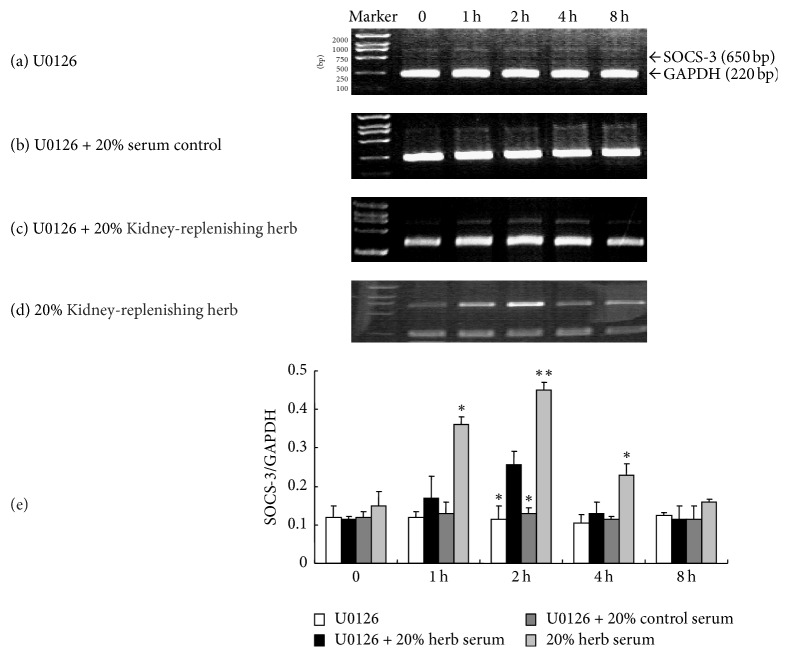
Kidney-replenishing herb induced SOCS-3 transcription of the first-trimester human in the presence of U0126 for 8 h. The results suggested that Kidney-replenishing herb could stimulate the SOCS-3 transcription of human trophoblast cells in a time-dependent manner, and the SOCS-3 induced by Kidney-replenishing herb could even be sustained at least for 4 h. U0126, as a MEK inhibitor, remarkably eliminated SOCS-3 transcription induced by Kidney-replenishing herb. Data shown are representative of three independent experiments. ^*∗∗*^*p* < 0.01, ^*∗*^*p* < 0.05 versus 20% control serum.

After starved with 1% FBS for 12 h, the primary cultured trophoblasts were treated with 20% Kidney-replenishing herb, 20% Kidney-replenishing herb or 20% control serum combined with U0126 (30 *μ*mol/L), and U0126 (30 *μ*mol/L) alone for 8 h, respectively. As shown in Figure 6, U0126 remarkably inhibited the SOCS-3 transcription induced by Kidney-replenishing herb in trophoblast cells (1 h, 4 h, and 8 h), except U0126 effect on the SOCS-3 expression to that of 20% Kidney-replenishing herb at 2 h (*p* < 0.05), shown in Figure 6(c). The results indicated that Kidney-replenishing herb has the potential to stimulate the ERK1/2/MAPK signaling to induce the transcription of SOCS-3 in human trophoblast cells (Figure 6(e)), but there is one else signaling to induce SOCS-3 transcription. Based on this finding, we next explored the effects of U0126 on the level of SOCS-3 protein expression in trophoblast cells. After treatment of trophoblast with U0126 for 20 min, we evaluated the expression of SOCS-3 in the presence of Kidney-replenishing herb (1, 2, 4, and 8 h), but we did not find the SOCS-3 protein by Western blot analysis. The experiment was performed in triplicate with essentially similar results. These results indicate that Kidney-replenishing herb can upregulate SOCS-3 expression via ERK1/2 pathway.

### 3.6. Kidney-Replenishing Herb Induced ERK1/2 Phosphorylation and SOCS-3 Protein Expression In Vitro in JAR Cells

**Figure 7:**
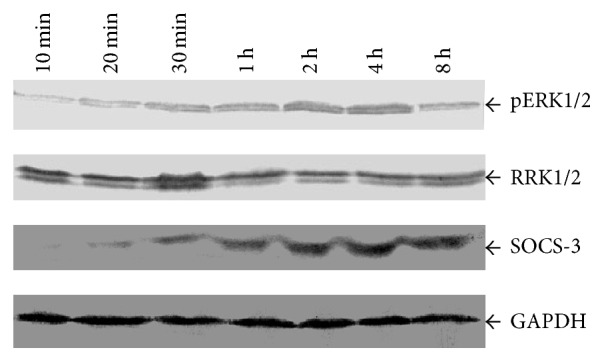
Kidney-replenishing herb induces phosphorylation of ERK1/2 and increases SOCS-3 protein. JAR cells were serum-starved for 24 h and stimulated with 10% Kidney-replenishing herb for 8 h. Western blot analysis was performed using antibodies against pERK1/2, ERK1/2, SOCS-3, and GAPDH.

After observing increased ERK1/2 phosphorylation and SOCS-3 expression in the first-trimester human trophoblast cells treated with Kidney-replenishing herb, we detected the pERK1/2 and SOCS-3 protein in the human placenta choriocarcinoma JAR cells by Western blot. After serum being deprived in DMEM for 24 h, JAR cells were cultured and then treated with 10% Kidney-replenishing herb. As shown in Figure 7. The phosphorylation of ERK1/2 was observed in response to herb treatment, and the maximal effect was achieved at 2 h and then slowly declined until 8 h herb stimulation. Expression of SOCS-3 was detected at 10 min and peaked at two hours in response to Kidney-replenishing herb treatments; the significant SOCS-3 protein amount was determined after 8 h herb treatment. The results suggested that Kidney-replenishing herb could also induce ERK1/2 phosphorylation and SOCS-3 protein expression in JAR cells.

### 3.7. Kidney-Replenishing Herb Induced ERK1/2 Phosphorylation after the SOCS-3 siRNA Interference in JAR Cells

**Figure 8:**
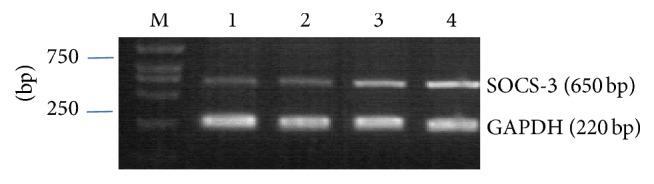
RT-PCR analysis of SOCS-3 mRNA expression after transfected with Stealth siRNA at 48 h. M: marker; lane 1, 2: cells transfected with Stealth siRNA; lane 3: cells transfected negative control; and lanes 4: untransfected cells. GAPDH expression was used as an internal control. Molecular weight is shown on the left.

**Figure 9:**
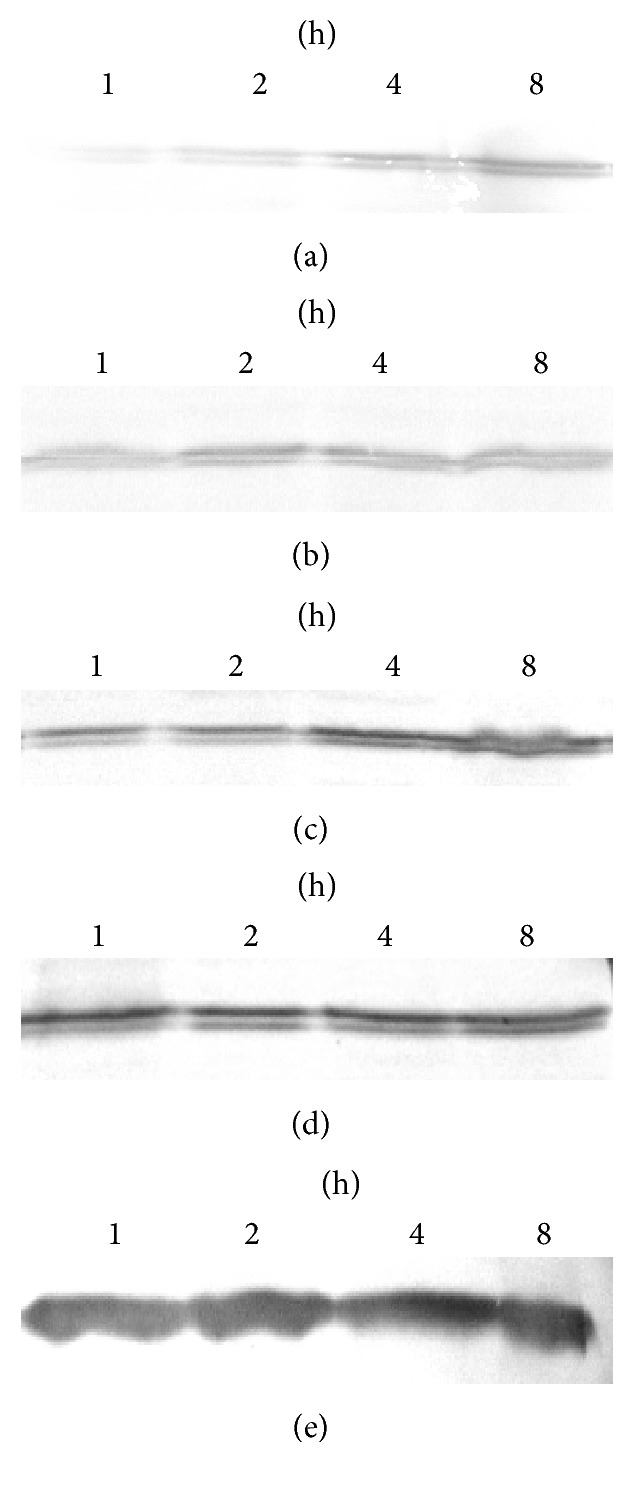
10% Kidney-replenishing herb-induced ERK1/2 phosphorylation was not affected by SOCS-3 interference in JAR cells. JAR cells were starved for 24 h, subjected to 10% Kidney-replenishing herb treatments for 8 h. The phosphorylation of ERK1/2 was observed in response to vehicle control (a) and 10% control serum (b), the protein levels of pERK1/2 was significantly increased after treatment with 10% Kidney-replenishing herb (c). The samples were controlled by immunoblot against ERK (d) and GAPDH (e).

In the present work, we aimed to study the influence of knockdown of SOCS-3 expression by RNA interference (siRNA) on the SOCS-3 expression and ERK1/2 phosphorylation in JAR cells. Cells transfected with BLOCK-iT vector only were used as a negative control. RT-PCR analysis showed that SOCS-3 mRNA in JAR cells was inhibited successfully by 70% (Figure 8), and Western blot analysis showed that SOCS-3 protein expression in JAR cells was silenced. We further investigated the effect of SOCS-3 silence on the ERK1/2 phosphorylation. After 48 h of transfection, pERK1/2 protein expression was still induced by exposure of cells to 10% Kidney-replenishing herb (1, 2, 4, and 8 h), pERK1/2 protein levels peaked at 4 h, and declined thereafter (Figure 9(c)), suggesting that ERK1/2 phosphorylation in trophoblast might be not dependent on SOCS-3 expression.

### 3.8. SOCS-3 Overexpression Enhanced Proliferation of JAR Cells

**Figure 10:**
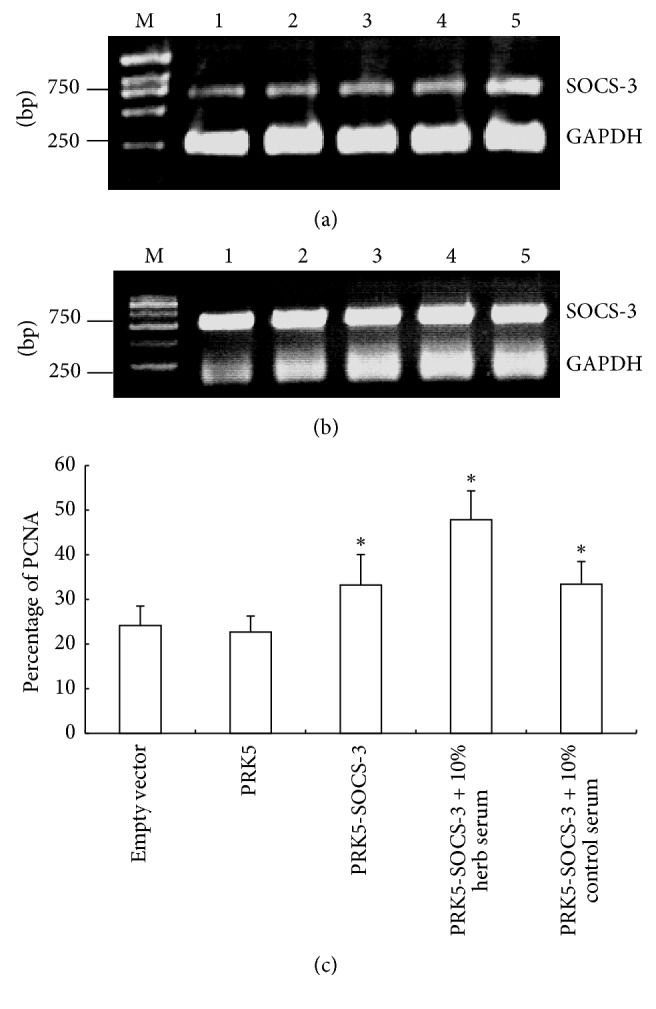
Transfection of SOCS-3 gene into JAR trophoblasts. RT-PCR analysis of SOCS-3 mRNA expression after treatment with 10% of control serum (a) and 10% Kidney-replenishing herb (b). M: marker; lane 1: SOCS-3 expression which was detectable in untransfected cells; lane 2: cells transfected with liposome; lanes 3: cells transfected with PRK5; lanes 4: cells transfected with pRNAT-U6, and lanes 5: cells transfected with SOCS-3-PRK51 at 48 h. (c) JAR cells proliferation was measured by using flow cytometry; trophoblast cells proliferation was increased after SOCS-3 overexpression. ^*∗*^*p* < 0.05 versus untransfected cells.

RT-PCR showed that SOCS-3 mRNA expression in JAR cells was increase after transfection SOCS-3-PRK5 plasmid for 48 h (Figure 10(a)). We further observed the SOCS-3 transcription increased obviously after treatment with 10% Kidney-replenishing herb (Figure 10(b)). Since Kidney-replenishing herb could stimulate human trophoblast proliferation, we further determined the influence of SOCS-3 overexpression on the cell proliferation in JAR cells. Treatment of JAR cells with serum starve for 72 h and JAR cells was transfected with PRK5 empty vector or SOCS-3 expression plasmid; the flow cytometry was carried out to measure the expression of PCNA. The percent of empty vector-transfected cells showed no increase in PCNA expression (Figure 10(c)), whereas cells transfected with SOCS-3 plasmid were 33.24 ± 6.82% (*p* < 0.05), suggesting that SOCS-3 overexpression might partially increase JAR cells proliferation. Furthermore, treatment with Kidney-replenishing herb stimulated JAR cells proliferation compared with the same concentration control serum after SOCS-3 overexpression (*p* < 0.05). Thus, it is likely that SOCS-3 expression might be involved in the proliferation of human trophoblasts.

## 4. Discussion

It is known that the placenta plays an important role in the successful pregnancy. The placenta supply nutrition to the growing fetus, of which development is vital for fetal survival and growth [15]. Placental function is essential for the development of the mammalian embryo during pregnancy [16]. Maintenance placental normal function depends on the perfect balance among trophoblast layer proliferation, maturation, and apoptosis [17]. There is evidence of predominant proliferation in normal human first-trimester villous placental trophoblast, especially in cytotrophoblast. Meantime, apoptosis is thought to play physiological roles in placental growth [18]; the rate of apoptosis is low throughout normal early pregnancy. The inadequate proliferation or excessive apoptosis is reported in human placenta complicated with a few abnormal pregnancies such as spontaneous abortion, preeclampsia, preterm delivery, and intrauterine growth restriction [19–21]. For this reason, how to enhance the biological functions of trophoblast cells is of great interest for researchers on reproductive medication.

Kidney-replenishing herb is a traditional drug which widely used to treat spontaneous abortion. Our previous studies demonstrated that Kidney-replenishing herb can modulate the balance of Th1/Th2 cytokines at the maternofetal interface and maintains pregnancy by accelerating its growth and fusion and reducing the rate of apoptosis [14]. Our present study founded that exposure of serum-starved normal human trophoblast cells to Kidney-replenishing herb for 48 h resulted in a dose-dependent promotion of cell proliferation activity; proliferating cell nuclear antigen (PCNA) expression was also induced by exposure of cells to herb. PCNA is a well-known cell-cycle marker that has been proved as a useful tool for determining the proportion of proliferating cells [22]. The cell cycle is divided into distinct phases in mammalian. Quiescent cells exist in the G0 phase; when they enter the cell cycle at G1, cells begin DNA replication, and PCNA expression increases quickly at the same time. If cells pass through the S phase, PCNA reached the peak as DNA replication is completed, and then cells progress through the G2 phase before entering mitosis (M-phase); meanwhile PCNA expression correspondingly decreases.

Our previous studies have showed that Kidney-replenishing herb reduced the percentage of normal human cytotrophoblast cells entering sub-G1-phase and shifted cell-cycle phase from G1 phases to S phase. In agreement with our previous finding, our study found that Kidney-replenishing herb can promote PCNA expression of human first-trimester trophoblast in a dose-dependent manner. Furthermore, we have assayed the early phase of apoptosis, triggered by serum deprivation, by using Annexin V-propidium iodide (PI) labeling and flow cytometric analysis. Kidney-replenishing herb showed significant decrease apoptosis of primary trophoblasts in this study. In conclusion, our study observed that administration with Kidney-replenishing herb promoted proliferation and diminished cell apoptosis of human first-trimester trophoblasts, even in the presence of U0126. However, less is known about how Kidney-replenishing herb controls trophoblast cell proliferation and apoptosis.

Cytokines and growth factors regulate the growth and differentiation of cells by binding to cell-surface receptors and activating intracellular signal transduction cascades. The suppressors of cytokine signaling (SOCS) family of proteins (SOCS-1~SOCS-7 and CIS) regulate cytokine signaling. SOCS-3 is regulated in multiple cell types and induced by IL-6, IL-11, leptin, LIF, and so on [23]. Many reports confirmed that SOCS-3 have profound effects on the regulation of immunity and inflammation by affecting the activation, development, and homeostatic functions of all lineages involved in immune and inflammatory responses. SOCS-3 expression also has been detected in trophoblast cells. Moreover, it seems that SOCS-3 may be involved in the process of placentation.

SOCS-3 knockout gene in the mouse presents several placental anomalies such as poorly formed spongiotrophoblast and labyrinthine layers and is accompanied by increased numbers of mature trophoblast giant cells. These several placental insufficiencies ultimately result in mice which die during the embryonic stage of development; however, these embryos can be saved by a tetraploid rescue approach [11, 12, 24]. These observations gave some useful clues for exploring the effect of SOCS-3 on human trophoblast cells.

Lee and colleagues reported cytoplasmic and nuclear expression of SOCS in vitro and SOCS-3 nuclear translocation only when its levels increase [10]. White et al. demonstrated that none of SOCS-3 expression was found in the nucleus, while little SOCS-3 expression was found in cytoplasm [25]. Our data demonstrated cytoplasmic and nuclear localization of SOCS proteins in the human placenta of the first trimester of pregnancy by immunohistochemical analyses in vivo, suggesting there are sufficient expressions of SOCS-3 in placental trophoblasts which play a crucial role in regulating placenta function. Our data further demonstrated that SOCS-3 is expressed in purified human trophoblasts and JAR trophoblast cell line by RT-PCR and Western blot. According to Goren and colleagues, SOCS-3 has a novel role as a regulator of keratinocyte proliferation and differentiation in vitro and in vivo [26]. We founded that Kidney-replenishing herb to human trophoblast cells showed a dose-responsive stimulation of proliferation. Meanwhile, treatment with Kidney-replenishing herb induced SOCS-3 mRNA expression in trophoblast in a dose-dependent manner. We observed that SOCS-3 overexpression increases basal proliferation of JAR. Thus, it was reasonable to guess that SOCS-3 may be related to the trophoblastic proliferation and the development of placenta. We further observed that signaling pathways induced SOCS-3 in order to find its precise physiological function.

The mitogen-activated protein kinase (MAPK) is one of revolutionary signaling pathways in mammalian cells including the extracellular signal-regulated kinases (ERK1/2), the p38 MAP kinases (p38a, p38b, p38g, and p38d), the c-Jun-NH2-terminal kinases (JNK1, JNK2, and JNK3), and ERK5 [27]. The activation of the ERK signaling pathway has been associated with cell proliferation, differentiation, and cell survival and provides protection against apoptosis [8]. It is well known that ERK1/2/MAPK cascade is one of the major signaling pathways involved in placenta development. ERK1/2 is also widely expressed in villous cytotrophoblasts in the first-trimester gestation [28]. The proliferation of trophoblast cells is dependent on activation of ERK 1/2, and disruption of the ERK2 locus leads to embryonic lethality early in mouse development after implantation [8]. Our study showed that Kidney-replenishing herb could induce the activation of ERK1/2 in a dose-dependent manner and promote the growth of trophoblast cells in vitro. U0126, a MEK inhibitor, could significantly inhibit this enhanced phosphorylation of ERK1/2 and proliferation of trophoblast cells induced by Kidney-replenishing herb. In addition, we have found that Kidney-replenishing herb prevents the apoptotic process by means of the activation of ERK pathway. It suggested that Kidney-replenishing herb promotes proliferation and prevents the early events of apoptosis via ERK pathway in human trophoblasts. On the other hand, we found that Kidney-replenishing herb increased SOCS-3 expression and activated the ERK1/2 pathway; U0126 could significantly abrogate the mRNA and protein expression of SOCS-3 induced by Kidney-replenishing herb in trophoblast cells. It indicated that Kidney-replenishing herb enhanced SOCS-3 expression through activation of the ERK1/2 pathway in trophoblast cells. Several studies demonstrated that the activation of ERK1/2 is involved in the regulation of SOCS-3 expression in response to cytokine [29, 30]. However, the mechanisms governing the ERK/MAPK-dependent regulation of SOCS-3 expression are not well understood. We hypothesized that Kidney-replenishing herb may affect trophoblast proliferation by regulating SOCS-3 expression. To confirm the role of SOCS-3 expression in herb-induced ERK pathway, JAR human choriocarcinoma cells are used to study cellular signaling, which has the characteristics of early placental trophoblast cells [31]. We knocked down SOCS expression by RNA interference (RNAi) in JAR cells and reevaluated the effect of herb on activation of ERK pathway. Our data showed that ERK phosphorylation stimulated by Kidney-replenishing herb has no change by SOCS-3 siRNA silence. We further founded that the SOCS-3 overexpression in JAR trophoblast cells triggers the proliferation of themselves. Thus, it is proposed that the intracellular ERK phosphorylation is an upstream signal of SOCS-3 expression, and Kidney-replenishing herb enhanced SOCS-3 expression through activation of the ERK1/2 pathway, thereby inducing trophoblast proliferation.

Collectively, our findings imply that the stimulatory effect of Kidney-replenishing herb on trophoblast proliferation and SOCS-3 expression depends on the ERK1/2 pathway, and SOCS-3 may be involved placentation and embryonic development. Interestingly, U0126 only partly suppressed SOCS-3 mRNA and proliferation of the trophoblasts upregulated by Kidney-replenishing herb, suggesting that, besides ERK signals, there might be other signal pathways which were also involved in the Kidney-replenishing herb-induced growth of human trophoblasts.

SOCS-3 have been identified as important regulators of ERK/MAPK pathway and STAT3 signaling in undifferentiated Rcho-1 cells, which were derived from rat choriocarcinoma [12]. Other studies showed that STAT3 signal transduction pathway induces SOCS-3 in acute myeloid leukemia cells [32] and in rat astrocytes [33]. We wonder if Kidney-replenishing herb upregulates SOCS-3 expression through activation of STAT3 pathway in human trophoblast cells. Because Kidney-replenishing herb in cytotrophoblasts could promote the expression of SOCS-3 in the first-trimester human trophoblast at one-hour stimulation, we detected the phosphorylation of STAT3 in response to herb treatment in first-trimester human trophoblast cells at 10 min, 20 min, 30 min, and one hour, respectively; however we did not detect any phosphorylation of STAT3 (data not shown). It suggests that STAT3 pathway is not involved in the regulation of SOCS-3 expression induced by Kidney-replenishing herb. Huang and colleagues study also indicates that there was SOCS-3 expression but no STAT3 activation by IFN-*γ* at one hour [34]. Other data show that there is an inverse correlation between the expression of SOCS-3 and IL-6-induced phosphorylation of STAT3 in prostate cancer cells; furthermore, downregulation of SOCS-3 by a short interfering RNA approach resulted in inhibition of proliferation and an increased apoptotic rate [35]. It is not known whether various types of STAT3 expression relative to SOCS-3 might be different stimulation or different cultured cells.

Many publications suggested that STAT3 and ERK/MAPK pathways are involved in the regulation of cell proliferation, apoptosis, and angiogenesis and participants in the processes of induction, progression, and metastasis of carcinoma. Activation of STAT3 may prevent death of tumor cells and stimulates tumor proliferation, invasion, and migration [35–37]. It is possible that the activation of ERK-1/2 but not STAT3 which was induced upon Kidney-replenishing herb treatment in trophoblast is the mechanism that herb regulates trophoblast cell proliferation and avoids its overgrowth.

## 5. Conclusions

Our study has shown that Kidney-replenishing herb might promoted survival and antiapoptosis via ERK1/2 signaling pathway and provides the first evidence for SOCS-3 in the regulation of human first-trimester trophoblast. Further work on Kidney-replenishing herb will need to be done, which could be useful in therapeutics of recurrent spontaneous abortion and other pregnant complications. It could be inferred that elucidating the role of SOCS-3 in ERK1/2 pathways might provide clues as to how trophoblast cells can be controlled by Kidney-replenishing herb, as well as insight into how SOCS-3 regulates survival and growth in trophoblasts.
